# Identification of Spiro-Fused [3-azabicyclo[3.1.0]hexane]oxindoles as Potential Antitumor Agents: Initial In Vitro Evaluation of Anti-Proliferative Effect and Actin Cytoskeleton Transformation in 3T3 and 3T3-SV40 Fibroblast

**DOI:** 10.3390/ijms22158264

**Published:** 2021-07-31

**Authors:** Nickolay A. Knyazev, Stanislav V. Shmakov, Sofya A. Pechkovskaya, Alexander S. Filatov, Alexander V. Stepakov, Vitali M. Boitsov, Natalia A. Filatova

**Affiliations:** 1 Saint-Petersburg Clinical Scientific and Practical Center for Specialized Types of Medical Care (Oncological), 197758 Saint Petersburg, Russia; 2 Institute of Cytology, Russian Academy of Sciences, 194064 Saint Petersburg, Russia; sapechkovskaya@gmail.com (S.A.P.); n_filat@mail.ru (N.A.F.); 3 Saint Petersburg National Research Academic University of the Russian Academy of Sciences, 194021 Saint Petersburg, Russia; stas-svs@list.ru; 4 Department of Chemistry, Saint Petersburg State University, 199034 Saint Petersburg, Russia; shurikfilatov@yandex.ru (A.S.F.); alstepakov@yandex.ru (A.V.S.); 5 Scientific and Research Centre, Pavlov First Saint Petersburg State Medical University, 197022 Saint Petersburg, Russia

**Keywords:** actin, metastasis, 3T3, 3T3-SV40, 3-spiro[azabicyclo[3.1.0]hexane]oxindoles, tumor cells, ADMET analysis, cytoskeleton, cell cycle

## Abstract

Novel heterocyclic compounds containing 3-spiro[3-azabicyclo[3.1.0]hexane]oxindole framework (**4a**, **4b** and **4c**) have been studied as potential antitumor agents. The in silico ADMET (adsorption, distribution, metabolism, excretion and toxicity) analysis was performed on **4a**–**c** compounds with promising antiproliferative activity, previously synthetized and screened against human erythroleukemic cell line K562 tumor cell line. Cytotoxicity of **4a**–**c** against murine fibroblast 3T3 and SV-40 transformed murine fibroblast 3T3-SV40 cell lines were evaluated. The **4a** and **4c** compounds were cytotoxic against 3T3-SV40 cells in comparison with those of 3T3. In agreement with the DNA cytometry studies, the tested compounds have achieved significant cell-cycle perturbation with higher accumulation of cells in G0/G1 phase. Using confocal microscopy, we found that with **4a** and **4c** treatment of 3T3 cells, actin filaments disappeared, and granular actin was distributed diffusely in the cytoplasm in 82–97% of cells. The number of 3T3-SV40 cells with stress fibers increased to 7–30% against 2% in control. We discovered that transformed 3T3-SV40 cells after treatment with compounds **4a** and **4c** significantly reduced the number of cells with filopodium-like membrane protrusions (from 86 % in control cells to 6–18% after treatment), which indirectly suggests a decrease in cell motility. We can conclude that the studied compounds **4a** and **4c** have a cytostatic effect, which can lead to a decrease in the number of filopodium-like membrane protrusions.

## 1. Introduction

Cancer is one of the fatal diseases, which is characterized by uncontrolled cell division leading to metastasis and invasion into adjacent tissues. Synthesizing new anticancer agents with less side effects and increased selectivity is still a developing area of interest in medicinal chemistry [1]. A significant portion of the improved clinical outcomes in cancer can be attributed to refined chemotherapeutic agents. The identification of antitumor natural products and natural product analogs such as Paclitaxel, Vinblastine, Doxorubicin, and Hycamtin has clearly demonstrated the importance of natural products in modern medicine [2].

Nowadays, many potential drugs fail to reach the clinic because of in silico prediction liabilities. Adsorption, distribution, metabolism, excretion and toxicity (ADMET) processes play a pivotal role in defining the therapeutic efficacy of drugs. Drug likeness appears as a promising paradigm of a compound that optimizes their ADME in the human body [3].

Spirooxindole alkaloids, as a family member of oxindole natural products, were first isolated from roots of Gelsemium sempervirens (wild yellow jasmine). Additional oxindoles were isolated from Aspidosperma, Mitragyna, Ourouparia, Rauwolfia and Vinca [4]. The privileged scaffold of spirooxindole contains two basic substructure units: One is multiple functionalized oxindole, which can be used as hydrogen bond donors and acceptors to interact with biological targets, and the other is a cycloalkyl or heterocyclic moiety fused at the C-3 position of oxindole. Accordingly, the unique spatial architecture and significant biological activities of spirooxindole have long captured the great attention of researchers [5,6]. Along with the molecules of natural origin based on 3-spirooxindoles possess pronounced biological activity such as anti-inflammatory, antibacterial and antifungal capacity [7,8]. Earlier, we developed an efficient method for the synthesis of various functionally substituted heterocyclic compounds containing spiro-fused oxindole, tryptanthrin, ninhydrin or 11*H*-indeno[1,2-*b*]-quinoxalin-11-one fragments with azabicyclo[3.1.0]hexane or cyclopropa[*a*]pyrrolizine moiety, by 1,3-dipolar cycloaddition of different cyclopropenes to in situ generated azomethine ylides [9,10,11,12]. All synthesized compounds led to a decrease in the growth rate of human erythroleukemic cells, K562. However, the effect of novel synthetized compounds on the cytoskeleton and cell motility is still unknown.

In this regard, we have chosen the model of immortalized 3T3 murine fibroblasts and 3T3 transformed with the SV40 virus (3T3 SV40) ones to study their response (including cytoskeleton) on the action by synthesized compounds. This model is described in the literature and is widely used to study the resistance of mammalian cells to bacteria and the action of various chemicals, including antioxidants, in normal and pathological conditions [13]. Moreover, these cell lines are widely used to study the structure of the cytoskeleton [14,15]. It was shown that under the action of N-acetylcysteine, there is a change in the actin cytoskeleton structure in 3T3-SV40 typical to normal cells [16]. These cell lines have a pronounced actin cytoskeleton, which is involved in the formation of lamellapodia, pseudopodia and filopodia that take an active part in cell motility. Therefore, the chosen model will allow to evaluate the effect of the studied compounds on the actin cytoskeleton changes and make the predictions on cell motility potential.

Due to the fact that spiro-fused [azabicyclo[3.1.0]hexane]oxindoles showed potential cytotoxic activity against a number of tumor cell lines, the aim of this study was to evaluate the potential antitumor activity of synthetized compounds as potential pharmacological agents. The objectives of the work were to test synthetized compounds for drug likeness in silico, to evaluate their effect on growth rate, cell cycle and actin cytoskeleton transformations in a model of immortalized and transformed fibroblasts 3T3 and 3T3 SV40 and compare their effect with a well-known antitumor agent.

## 2. Results and Discussion

### 2.1. Drug-Likeness and Bioactivity Scores

Based on the aim of estimating the drug-likeness of the compounds **4a**–**c**, we have determined the compliance of the synthesized molecules to the Lipinski rule of five. According to this rule, poor absorption or permeation is more likely when there are more than five hydrogen bond donors, ten hydrogen bond acceptors, the molecular weight is greater than 500 g/mol and the calculated log p (logarithmic ratio of the octanol–water partitioning coefficient) is greater than 5. Molecules violating more than one of these parameters may have problems with bioavailability and a high probability of failure to display drug-likeness [15]. Further, the topological polar surface area (TPSA), which is another key property in estimating drug bioavailability, was also calculated. TPSA is fragment-based methodology, which derived standardized contributions to the molecular polar surface area from functional groups and atom types [17]. Generally, compounds with a TPSA > 140 Å2 are thought to have low bioavailability [18] and tend to be poor at permeating cell membranes [19]. For molecules to penetrate the blood–brain barrier (and thus affect the receptors in the central nervous system), a TPSA less than 90 Å2 is usually needed [20]. 

A number of ADME parameters such as molecular weight, number of hydrogen bond donors, number of hydrogen bond acceptors, logarithmic ratio of the octanol–water partitioning coefficient (LogP) and topological polar surface area were calculated for the compounds **4a**–**c** in silico according to the Lipinski rule of five. All candidates having LogP in the range of 5.96–6.88 and molecular weight in the range of 484.64–502.68 g/mol indicate good bioavailability with less than 10 freely rotatable bonds. The TPSA of all the derivatives is in the range of 41.12–41.14 Å2 shows their ability to permeate cell membranes. In this study, all the derivatives exhibited fewer than 5 hydrogen bond donors (Table 1). 

Different ADME parameters were also predicted for all compounds. The obtained ADME predictions (Table 2) indicated that all the compounds showed strong binding to plasma proteins (PPB, more than 97%) and high human intestinal absorption values (HIA, 95.5–96.7%), while Caco-2 cell permeability ranged from 23.9 to 48.3 nm/s. Additionally, according to the predictions, the latter were found to have moderate penetration to the central nervous system through blood–brain barrier (BBB, 0.9–8.6). It can be concluded, therefore, that, theoretically, compounds **4a**–**c** can show good absorption and bioavailability with reasonable blood–brain barrier permeability.

It is obvious from Table 1 and Table 2 that all of the synthesized compounds have parameters that are close to those characterizing drug-likeness. The selected substances are similar in most of the parameters except for logP, penetration through the BBB and penetration of the Caco2 cells through the cell membrane. Therefore, according to the in silico predictions, substance **4c** should be the worst at penetrating the blood brain barrier (0.99 versus 8.8) and twice as bad at penetrating through the cell membrane. However, according to parameters such as TPSA and logP, no significant differences were observed. Therefore, compounds **4a**–**c** demonstrated good properties according to Lipinski’s ‘rule of five’ and, accordingly, are promising targets for further research in vitro.

### 2.2. Cell Multiplication Study after ***4a**–**c*** Compounds Treatment

**Figure 1:**
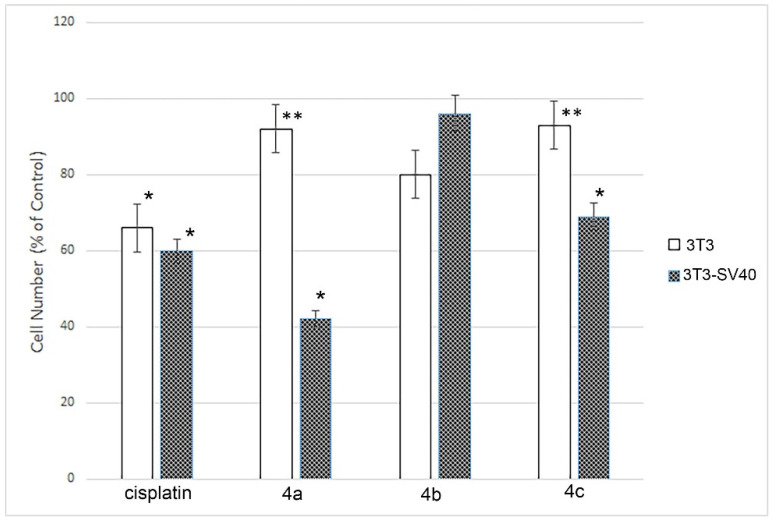
Effect of spiro-fused [3-azabicyclo[3.1.0]hexane]exindoles derivatives **4a**–**c** and cisplatin on growth 3T3 and 3T3 SV40 cells. The graph demonstrates the effect of the compounds on cell growth in relation to the control. Results are averages of 3 replicates in each experiment, given as the mean ± SD values. * *p* ≤ 0.05. ** intergroup significance levels *p* ≤ 0.05.

Flow cytometry revealed that treatment with compounds **4a**–**c** significantly decreased multiplication capacity of only 3T3-SV40 cells. In controls (without treatment), the proportion of dead cells was very low, not exceeding 2%. The growth rate of the untreated cells populations (controls) of highly transformed 3T3-SV40 cells was 1.7 times higher than in the immortalized 3T3 cells after 24 h. Figure 1 demonstrates that the compounds **4a**–**c** treatment of 3T3 cells did not lead to a growth inhibition of the cells, while cisplatin significantly slowed it down (1.6 times). The effects of **4a** and **4c** substances on 3T3-SV40 cells resulted in the inhibition of cell growth to the effect level of cytostatic drugs like cisplatin (1.7 times): in the case of **4c** it was 1.5 times, and even more significant for **4a** (2.4 times). And only one of the three studied substances (**4b**) did not change the growth rate of those cells significantly. It was shown that cisplatin inhibits the growth rate of both 3T3 and 3T3- SV40 (1.6, and 1.7 times, respectively). At the same time, none of the studied drugs affected the growth of immortalized 3T3 cells. The growth rate of highly transformed 3T3-SV40 cells was inhibited only by drugs **4a** and **4c** but not by **4b**.

Compounds **4a** and **4c** lead to the inhibition of cells with high growth rate potential (3T3-SV40), while growth retardation of cells with low growth rate potential (3T3) does not occur. Cisplatin, in turn, slows down the growth of cells with both low and high growth rate potential.

It is known that compounds based on spirooxindoles inhibit the growth of tumor cells such as adenocarcinoma MCF-7, cervical carcinoma HeLa, and prostate cancer DU-145 at concentrations from 2 to 20 mM depending on the cell line, which is comparable with our data [21].

All synthetized compounds consist of oxindole and 3-azabicyclo[3.1.0]hexane structural fragments with different substituents (including amino acids residues). It can also be noted that compounds with an alkyl or mercaptoalkyl group, isobutyl (**4a**), sec-butyl (**4b**) and 2-methylthioethyl (**4c**), showed the highest activity among the compounds with a spiro[azabicyclo[3.1.0]hexane]oxindole framework. It is also known that when the alkyl group is replaced by an amidocarboxylic and phenyl by a carboxymethyl group, these compounds lose their activity [9]. Earlier, we studied the influence of compounds attached to 3-azabicyclo[3.1.0]hexane fragment on erythromyelosis leukemia K562 cell line growth rate. It was shown that substances with phenyl substituents only lead to inhibition of cell proliferation of human erythromyelosis leukemia K562. Two groups of compounds included glycine and proline residues in their structures. However, these compounds did not lead to a change in the growth rate of K562 cells. In the case of alpha amino acids, the opposite situation was observed—proliferation of K562 was significantly reduced. Therefore, substances containing phenyl substituted cyclopropane ring with phenyl radicals and alpha-amino acid residues are most active against K562 cells [9].

### 2.3. Cell Cycle Analysis

**Figure 2:**
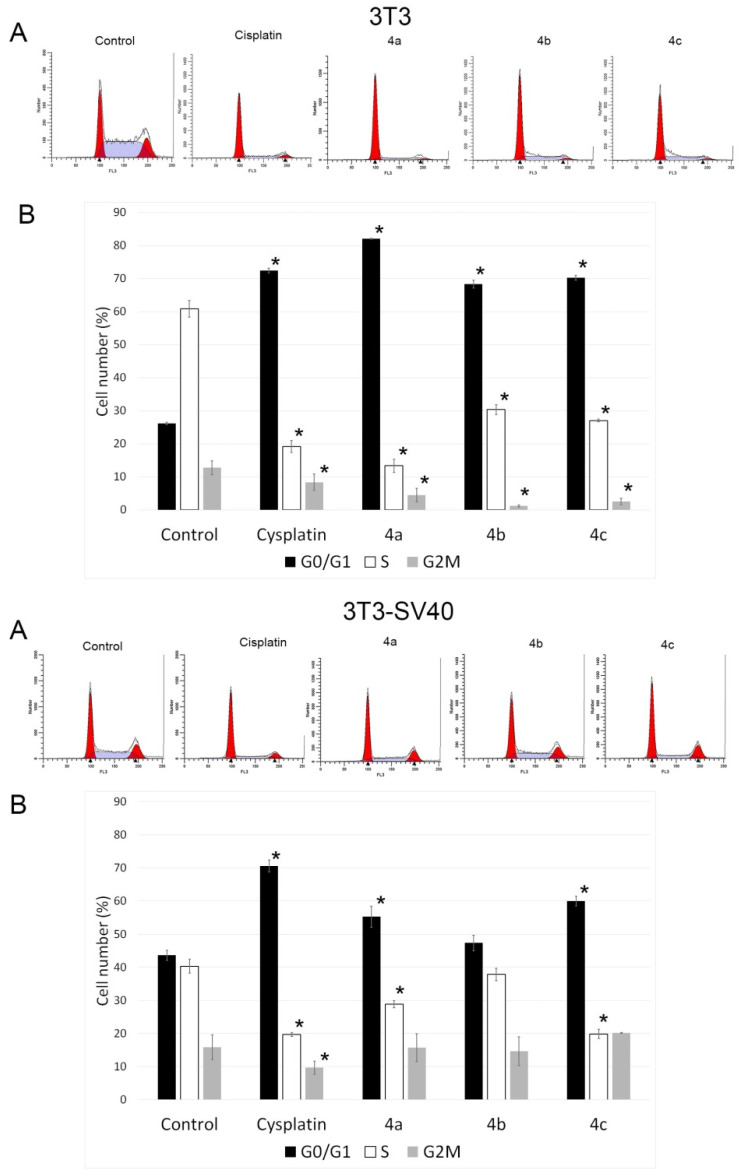
Effect of spiro-fused [3-azabicyclo[3.1.0]hexane]exindoles derivatives **4a**–**c** and cisplatin on the distribution of 3T3 and 3T3 SV40 cells in the cell cycle. The most typical histograms (**A**) of distribution of cells in the cell cycle are presented (3T3A and 3T3 SV40A). The graphs 3T3B and 3T3 SV40B (**B**) demonstrate the average distribution of the cells in the cell cycle based on 3 replicates in each experiment, given as the mean ± SD values. * *p* ≤ 0.05.

One of the indicators of the impact of various biologically active substances on the cells is the changes in the cell cycle. In this study, all synthesized compounds were examined for assessment of the possible distribution of cells in the cell cycle (G0/G1, G2/M and S) using flow cytometry. Figure 2 shows typical cytometric results (3T3A and 3T3-SV40A) and data after processing the results for three replicates of each experiment (3T3B and 3T3-SV40B). 

The analysis of the experimental results showed that all three drugs and cisplatin stop the 3T3 cell cycle at the G0 phase (or at the G0/G1 phase boundary) and slow down the exit of cells from the G2 phase (which can be concluded from the change in the ratio of the G2/M and S phases). Therefore, following the impact of drugs **4a**–**c**, the percentage of cells in the synthetic phase (S) of the cycle decreases to 13.4, 30.4, and 27.1%, respectively, against 60.9% in the control. The strongest cytostatic effect was observed after treatment with **4a** compound. The number of cells in the S phase was 15.7% and 82.2% in the G0/G1 phase versus 18% and 71.7%, respectively, under the impact of cisplatin. **4a** blocked the transition of cells to S-phase; the proportion of cells in the S-phase in this case was 15.7%, cells accumulated in the G0/G1 phase (82.2% versus 26.7% in the control), and their number decreased in the G2/M phase (2.1% versus 15.2% in the control). 

Treatment of 3T3-SV40 cells with the tested compounds resulted in suppression of cell proliferation, as in 3T3 cells. However, their delay in the G0 phase of the cell cycle (or at the G0/G1 phase boundary) and a decrease in the proportion of cells in the synthetic phase S were observed only under the action of drugs **4a** and **4c**, but not **4b**. Therefore, after the impact of compounds **4a** and **4c**, the number of cells in the synthetic phase S of the cycle decreased to 28.9%, and 19.9%, respectively, against 40.4% in the control and 19.7% under the action of cisplatin. The main difference in the sensitivity of 3T3-SV40 cells from 3T3 cells to the effect of the studied compounds was the absence of a change in the number of cells in the G2/M phase relative to the control and only a slight decrease in their number when impacted by cisplatin.

These results suggest that compounds **4a** and **4c** arrest 3T3 and 3T3-SV40 cells at the G0/G1 stage of the cell cycle. Based on the data given above, we can conclude that compounds **4a** and **4c** have the highest efficiency in terms of inhibition of the cell cycle and a decrease in the growth rate of highly transformed 3T3-SV40 and are comparable to the effect of cisplatin. Compound **4b** led to inhibition of the cell cycle only in the immortalized 3T3 cells. Since 3T3 and 3T3 SV40 cells had different growth rate potential in the control, and treatment with compounds **4a** and **4c** inhibited the cell cycle in both cases, and we can conclude that these two compounds have the highest efficiency.

In our work, it was shown that **4a**–**c** compounds stop the cell cycle at the G0/G1 phase, while in other studies it was shown that cell cycle arrest can occur in both the G0/G1 and G2M phases [21,22]. It is known that cisplatin, which is used as a reference drug, leads to the cell cycle’s arrest in most cell types in the S or G2 phases [23,24]. However, in the case of 3T3 mouse embryonic fibroblasts, the accumulation of cells under the action of cisplatin occurs in the G0/G1 phase [25], which is consistent with the data obtained in our work. The action of substances **4a**–**c** is similar to the effect of the reference substance cisplatin, which confirms that the investigated compounds also possess cytostatic activity.

### 2.4. Actin Cytoskeleton

**Figure 3:**
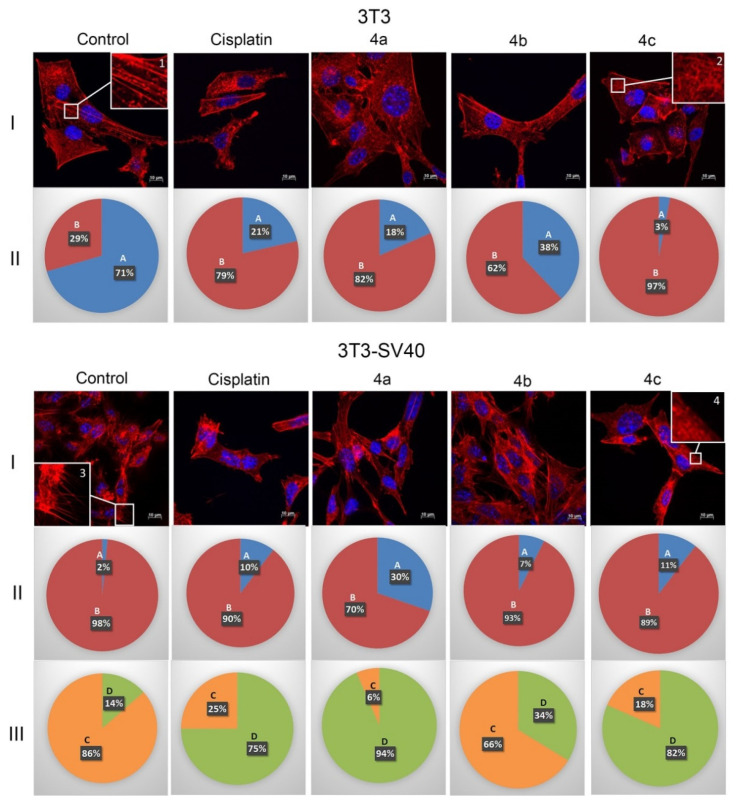
State of actin cytoskeleton of 3T3 and 3T3-SV40 cells after treatment with spiro-fused [3-azabicyclo[3.1.0]hexane]exindole derivatives **4a**–**c** and cisplatin. I: Images demonstrate the different stages of cell actin cytoskeleton (I-3T3 and I-3T3-SV40). II: Pie charts demonstrate percentage of cells with normal stress fibers (A) and disassembled stress fibers (B) (II-3T3 and II-3T3-SV40). III: Pie charts demonstrate percentage of cells with filopodia-like deformations (C), and without filopodia-like deformations (D). Inserts: 1—stress fibers; 2—disassembled stress fibers; 3—filopodia-like deformations; 4—without filopodia-like deformations.

The structure of the actin cytoskeleton of 3T3 and 3T3 SV40 cells is significantly different. 3T3 cells are characterized by the presence of actin stress fibers and the absence of filopodia-like deformations, while 3T3 SV40 cells are characterized by the absence of stress fibers and the presence of filopodia. Therefore, in this study, the structure of the actin cytoskeleton was assessed by the availability of stress fibers in 3T3 and 3T3 SV40 cells and the presence of filopodia-like protrusions after the impact of compounds **4a**–**c** (Figure 3).

3T3 cells were characterized by pronounced stress fibers in 71% of the cells, and microfilaments were almost completely disassembled in 29% of the cells (Figure 3 control 3T3, I (inserts 1 and 2) and II). Figure 3 demonstrates that the cisplatin treatment of 3T3 cells led to a decrease in the number of cells with stress fibers to 21% (against 71% in the control). The least effective of the compounds was **4b**. Although, it led to a decrease in the number of cells with stress fibers to 38%. Compound **4a** exhibited an effect, which was similar to the impact of cisplatin and led to a decrease in the number of cells with stress fibers to 18%. The most effective was **4c** compound, which reduced the number of cells with stress fibrils to 3%, that is, it was 7 times more effective than cisplatin. Thus, the investigated compounds **4a**–**c** affected 3T3 cells similarly to cisplatin, increasing the number of cells with disassembled stress fibers.

3T3 SV40 cells were characterized by the presence of stress fibers only in 2% of the cells, and in 98% of cells microfilaments were almost completely disassembled (Figure 3 control 3T3 SV40, I (Inserts 1 and 2) and II). The action of the drugs **4a**–**c** on 3T3-SV40 cells as well as cisplatin provides increasing the number of cells with stress fibers. Under the impact of compounds **4b** and **4c**, as well as under the action of cisplatin, the number of 3T3-SV40 cells with stress fibers increased to 7%, 11%, and 10%, respectively, and only after treatment with **4a** it increased up to 30%.

Therefore, the action of compounds **4a**–**c** leads to a decrease in the number of 3T3 cells with stress fibers, while the effect of these compounds on 3T3-SV40 cells leads to an increase in the number of cells with stress fibers.

The main difference between 3T3-SV40 cells and 3T3 cells is the presence or absence of filopodia-like deformations. We showed that in 3T3 cells, filopodia-like deformations were absent, but 86% of 3T3-SV40 cells had these structures. Compound **4b** led to the smallest decrease in number of cells with filopodia (up to 66%). Compound **4c** led to a decrease in the number of cells with filopodia to 18%, similarly to the effect of cisplatin, which led to a decrease in the number of cells with filopodia-like deformations to 25%. The most effective drug was **4a**, which reduced the number of cells with filopodia to 6%. We can conclude that in transformed 3T3-SV40 cells, all the studied drugs lead to a decrease in the population of cells with filopodia-like deformations, but the most effective is compound **4a** (Figure 3).

The obtained data allow us to consider the actin cytoskeleton as an additional target of antitumor chemotherapy [26,27]. Tumor transformation causes reorganization of the actin cytoskeleton, leading to change in cell motility. Correlation between the disappearance of actin stress fibers and increased migration activity of tumor cells was observed. The structural features of actin organization can serve as criteria for assessing the metastasis potential of tumor cells [28]. In this work, we studied the effects of synthesized **4a**–**c** compounds on the actin structure of 3T3 and 3T3-SV40 fibroblasts using fluorescence microscopy. It was shown that drug treatment using **4a**–**c** compounds led to significant changes of the actin cytoskeleton structure of tumor cells leading to the disappearance of stress fibers and changes in the number of filopodia-like deformations. 

There is a lack of cytoskeleton-specific research on spirooxindole derivates, but in the few works spirooxindole-containing compounds have been reported as actin polymerization inhibitors, and as inhibitors of tubulin polymerization [29,30]. Therefore, the actin cytoskeleton is a promising therapeutic target of new **4a**–**c** compounds.

## 3. Materials and Methods

### 3.1. In Silico Study

To predict the drug-likeness of selected compounds, the Lipinski rule of five was used [31]. This rule describes molecular properties important for a drug’s pharmacokinetics in a human body, including their absorption, distribution, metabolism and excretion (ADME). The Lipinski rule of five deals with simple physicochemical parameter ranges: Molecular weight (Mol. wt) ≤ 500, Octanol/water partition coefficient (C Log P) ≤ 5, H-bond donors ≤ 5 and H-bond acceptors ≤ 10. These parameters were calculated using Molinspiration online tool (https://www.molinspiration.com/cgi-bin/properties, accessed on 3 March 2021). Other ADME parameters such as Colon adenocarcinoma Caco2 permeability, Human intestinal absorption (HIA), Plasma protein binding ability (PPB) and ability to penetrate Blood–Brain-Barrier (BBB) were calculated using PREADMET online tool (https://preadmet.bmdrc.kr, accessed on 10 March 2021).

### 3.2. Synthesis

Compounds **4a**–**c** were prepared according to the procedure already successfully employed in our previous study [9]. 

General procedure for **4a**, **4b** and **4c** compounds synthesis. 

**Figure 4:**
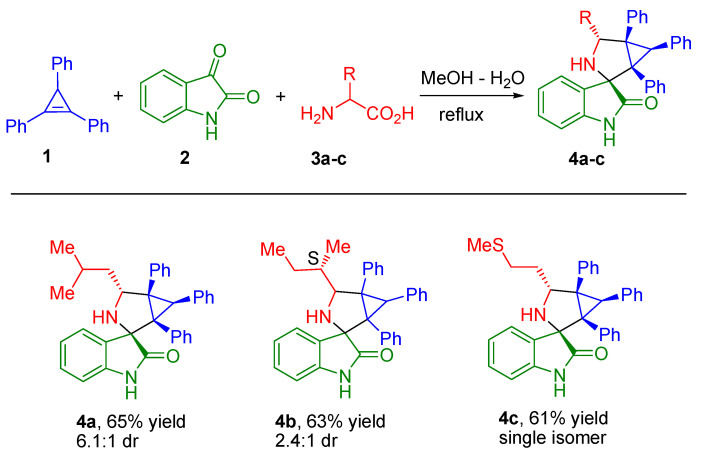
Synthesis of spiro-fused [3-azabicyclo[3.1.0]hexane]oxindoles **4a**, **4b**, and **4c**. Synthesized compounds containing spiro-fused 3-azabicyclo[3.1.0]hexane and oxindole moieties, by means of 1,3-dipolar cycloaddition reaction of appropriately substituted cyclopropene **1** to azomethine ylides generated *in-situ* from isatin **2** and corresponding α-amino acids **3a**–**c**.

A mixture of the cyclopropene **1** (0.2 mmol), isatin **2** (0.2 mmol), and corresponding α-amino acid **3a**–**c** (0.4 mmol) in a 3:1 mixture of MeOH−H2O (5 mL) was refluxed for 4−12 h; the reaction progress was monitored by thin layer chromatography. The solvent was evaporated under reduced pressure. The crude mixture was subjected to silica gel chromatography to obtain the pure products **4a**, **4b**, and **4c**; their structural formulas are presented in Figure 4.

### 3.3. Cell Culture and Culturing Conditions

Embryonic murine fibroblast Balb/3T3 (3T3) and fibroblast 3T3 transformed SV40 virus (3T3-SV40) were obtained from the Collection of Cell Cultures of Vertebrates (Institute of Cytology, Russian Academy of Sciences, St. Petersburg, Russia). Cells were cultivated in Dulbecco’s Modified Eagle Medium (DMEM) (Hyclone, USA) with addition of 10% fetal calf serum (Hyclone, Logan, UT, USA) and 40 mg/mL gentamicin (Sigma, St. Louis, MO, USA) at 37 °C with 5% CO_2_ in a humidified incubator.

### 3.4. Cell Treatment

To evaluate the cell growth potential and perform the cell cycle analysis, 3T3 and 3T3-SV40 fibroblasts were ceded at 24 well plates, 5 × 10^4^ cells per well. To evaluate changes in the actin cytoskeleton, 4 × 10^5^ 3T3 or 3T3-SV40 cells were ceded onto Petri dishes with cover glasses. The cells attached to the plates or cover glasses were treated with compounds **4a**–**c** or cisplatin at 25 μM concentration. The effect of the compounds was evaluated in 24 h after treatment.

### 3.5. Evaluation of Cell Multiplication and Cell Cycle Studies by Flow Cytometry

To separate the dead cells from the live cell population, propidium iodide (Invitrogen, Waltham, MA, USA) was added to the cell suspension at a concentration of 0.05 mg/mL. After incubation for 5 min at room temperature, each sample was analyzed by EPICS XL flow cytometer (Beckman Coulter, Brea, CA, USA). Cells negative to propidium iodide were considered as live cells. 

We used flow cytometry to study the phases of the cell cycle. The distribution of cells in the G0/G1-, S- and G2/M-phases of the cell cycle was assessed by determining the relative DNA content in cells. DNA staining by fluorescent dye propidium iodide was used for visualization of the experimental results.

For the cell cycle analysis, the samples were washed three times with phosphate-buffered saline (PBS) and then 0.2 mg/mL saponin (Sigma, St. Louis, MO, USA), 0.25 mg/mL RNase (Sigma, St. Louis, MO, USA), and 0.05 mg/mL propidium iodide (Invitrogen, Waltham, MA, USA) were added to each sample. After incubation for 30 min at room temperature, the samples were analyzed by a flow cytometer EPICS XL (Beckmann Coulter, Brea, CA, USA). Data processing was performed using the ModFit LT software (Verity Software House, Topsham, ME, USA). The average values and their variations based on obtained results are given in the results section; the most typical diagrams with the values are presented.

### 3.6. Actin Cytoskeleton Staining

Cells were washed three times with PBS. The cover slips were transferred onto parafilm, fixed with 3.7% paraformaldehyde (Sigma, St. Louis, MO, USA), and rinsed three times with PBS. For visualization of actin microfilaments, cells were stained with rhodamine phalloidin (Invitrogen, Waltham, MA, USA) for 15 min at 37 °C in an atmosphere of 5% CO_2_. The preparations were rinsed three times with PBS, and embedded in Fluoroshield medium (Sigma, St. Louis, MO, USA). Intensity of staining of preparations was estimated using an AxioObserver Z1 confocal microscope (Carl Zeiss, Jena, Germany). In each experiment, at least 30 cells were imaged. Images were processed using ImageJ software [32].

### 3.7. Statistics

Statistical processing of results was performed using MaxStat 3.06 software (MaxStat Software, Germany). All data from the three independent experiments were used for measuring the means ± standard error (mean ± SE) that were compared using the Student’s *t*-test or nonparametric U-Wilcoxon-Mann-Whitney test. Differences among groups were considered significant at *p* ≤ 0.05.

## 4. Conclusions

In this study, we established the ability of new 3-spiro[3-azabicyclo[3.1.0]hexane]oxindole derivates **4a**–**c** to reduce the viability, influence the cell cycle and reduce the filopodia-like protrusions of the transformed cells 3T3-SV40, while immortalized 3T3 cells were less affected by the studied substances. Compounds with isobutyl (**4a**) and 2-methylthioethyl (**4c**) groups were shown to downregulate growth of highly transformed 3T3-SV40 cells (similarly to cisplatin, which inhibits the growth rate of both 3T3 and 3T3-SV40 cells), as well as arrest the cell cycle in the G0/G1 phase of both 3T3 and 3T3-SV40 cell lines, and lead to a decrease in the number of 3T3-SV40 cells with filopodia-like deformations. The effect of compounds **4a**–**c** leads to an increase in the population of 3T3 cells with disassembled stress fibers, while the population of 3T3-SV40 cells with stress fibrils increases. All obtained data make it possible to assume that the studied substances, not only possess cytostatic activity, but are also capable of inducing changes in the actin cytoskeleton structure of tumor cells, which opens up broad prospects for the study of their action in vivo.

## Figures and Tables

**Table 1:** Physicochemical properties of the synthesized compounds.

Compound	Mol. wt ^1^	H-Bond Donors ^2^	H-Bond Acceptors ^3^	C Log P ^4^	TPSA (Å2) ^5^
Rule	≤500	≤5	≤10	≤5	≤140
**4a**	484.64	2	3	6.88	41.14
**4b**	484.64	2	3	6.88	41.12
**4c**	502.68	2	3	5.96	41.12
Cisplatin	300,05	6	2	−4.58	55.28

^1^ Molecular weight, ^2^ Number of hydrogen bond donors, ^3^ Number of hydrogen bond acceptors, ^4^ Logarithmic ratio of the octanol–water partitioning coefficient (C Log P), ^5^ Topological polar surface area (TPSA).

**Table 2:** Prediction of pharmacokinetic properties of the synthesized compounds.

Compound	Caco2 ^1^ Permeability	HIA ^2^ (%)	PPB ^3^ (%)	BBB ^4^ (C_brain_/C_blood_)
Rule	≥4	≥70	≥90	≥0.4
**4a**	48.3202	95.542381	97.857647	8.62779
**4b**	48.3128	95.542356	97.212995	8.82546
**4c**	23.9496	96.734547	100.000000	0.992983

^1^ Colon adenocarcinoma permeability, ^2^ Human intestinal absorption, ^3^ Plasma protein binding, ^4^ Blood–brain barrier penetration.

## Data Availability

The data presented in this study are available on request from the corresponding authors.
